# Longitudinal assessment of an anti-stigma campaign related to common mental disorders in rural India

**DOI:** 10.1192/bjp.2018.190

**Published:** 2019-02

**Authors:** Pallab K. Maulik, Siddhardha Devarapalli, Sudha Kallakuri, Anadya Prakash Tripathi, Mirja Koschorke, Graham Thornicroft

**Affiliations:** 1 Deputy Director and Director of Research, Research and Development, George Institute for Global Health, India and Senior Research Associate, George Institute for Global Health, University of Oxford, UK and Associate Professor, Faculty of Medicine, University of New South Wales, Australia; 2 Research Fellow, Research and Development, George Institute for Global Health, India; 3 Research Assistant, Research and Development, George Institute for Global Health, India; 4 Lead Biostatistician, Research and Development, George Institute for Global Health, India; 5 Visiting Lecturer, Centre for Global Mental Health and Centre for Implementation Science Health Service and Population Research, Institute of Psychiatry, Psychology and Neuroscience, King's College London, UK; 6 Professor of Community Psychiatry, Centre for Global Mental Health and Centre for Implementation Science Health Service and Population Research, Institute of Psychiatry, Psychology and Neuroscience, King's College London, UK

**Keywords:** Stigma, India, community based, longitudinal analysis, mental health services

## Abstract

**Background:**

Stigma related to mental health and lack of trained mental health professionals is a major cause for an increased treatment gap, particularly in rural India. The Systematic Medical Appraisal, Referral and Treatment (SMART) Mental Health project delivered a complex intervention involving task sharing, an anti-stigma campaign and use of technology-based, decision-support tools to empower primary care workers to identify and manage depression, anxiety, stress and suicide risk.

**Aims:**

The aim of this article is to report changes in stigma perceptions over three time points in the rural communities where the anti-stigma campaign was conducted.

**Method:**

A multimedia-based anti-stigma campaign was conducted over a 3-month period in the West Godavari district of Andhra Pradesh, India. Following that, the primary care-based mental health service was delivered for 1 year. The anti-stigma campaign was evaluated in two villages and data were captured at three time points over a 24-month period (*N* = 1417): before and after delivery of the campaign and after completion of the health services delivery intervention. Standardised tools captured data on knowledge, attitude and behaviour towards mental health as well as perceptions related to help seeking for mental illnesses.

**Results:**

Most knowledge, attitude and behaviour scores improved over the three time points. Overall mean scores on stigma perceptions related to help seeking improved by −0.375 (minimum/maximum of −2.7/2.4, s.d. 0.519, *P* < 0.001) during this time. Loss to follow-up was 10%.

**Conclusions:**

The data highlight the positive effects of an anti-stigma campaign over a 2-year period.

**Declaration of interest:**

None.

Stigma can be understood as consisting of three key elements: a problem of knowledge (ignorance/misinformation), a problem of attitudes (prejudice) and a problem of behaviour (discrimination).^1^ Two key reviews have identified interpersonal contact and educational materials (especially for adolescents) as effective intervention strategies to address stigma.^2^^,^^3^ Stigma is a major cause for non-utilisation or under-utilisation of mental health services globally,^4^ and this is an even greater issue in low- and middle-income countries (LMICs) where there is little evidence from research on which interventions are effective.^5^ Earlier research from the current study population showed that – following an anti-stigma campaign – there were definite improvements in the attitude and behaviour scores, with lesser effects on knowledge scores;^6^ a finding that has also been previously reported.^2^ Perceptions about help seeking also improved significantly. Qualitative data showed that social contact (in the form of a video of a person with a mental disorder speaking about their experience) and a drama depicting domestic violence, its effect on mental health and benefits of help seeking were the two most effective intervention strategies identified by the population. Those results were based on a mixed-methods, pre–post assessment conducted immediately following a 3-month anti-stigma campaign which covered a rural adult population of about 2000 people in two villages in the south Indian state of Andhra Pradesh.

The aim of this article is to report the longitudinal assessment of this cohort whom we re-interviewed about 2 years after the baseline evaluation. We wanted to assess the sustained impact of the initial 3-month intensive anti-stigma campaign, specifically in the absence of any further interventions. The campaign was part of a larger study called Systematic Medical Appraisal, Referral and Treatment (SMART) Mental Health.^7^

## Method

SMART Mental Health was conducted in the West Godavari district in the south Indian state of Andhra Pradesh. The aims were to assess the feasibility, acceptability and preliminary effectiveness of a mobile technology-enabled model used by primary healthcare workers for the delivery of mental health services. The key components of this complex intervention focused on the delivery of mental health services for common mental disorders (CMDs) (stress, depression and suicide risk) and involved: conducting an anti-stigma campaign prior to the delivery of health services across the villages; training of primary healthcare workers, i.e. lay village health workers known as Accredited Social Health Activists (ASHAs), and doctors on evidence-based tools; developing a mobile technology-based, electronic decision-support system based on those tools to facilitate the work of the health workers and doctors; and developing a system that provided a mechanism to follow-up with people with identifiable CMD using cloud computing and voice messages delivered through an algorithm-based, interactive voice-response system that provided reminders to patients, health workers and doctors.^7^^,^^8^

SMART Mental Health was conducted across 42 villages, but this formal evaluation of the anti-stigma campaign was limited to only two villages^8^ that were selected based on the following parameters: distance of each village is <40 kilometres from the field office, eligible population in each village is of average size (~1500), each village has at least two village health workers (ASHAs) and each village is under a different primary health centre. The decision to select only two villages was driven by the availability of funds.

### Eligibility

The evaluation involved all eligible adults ≥18 years old, who provided informed written consent and were available for interview. Those unable to comprehend the study questions due to severe physical or mental illness were excluded.

### Study design

The initial evaluation of the anti-stigma campaign used a pre–post design and the post-intervention data collection was conducted immediately after the 3-month anti-stigma campaign. The anti-stigma campaign was delivered by the research team across the community. Following the campaign, the other components of the intervention were rolled out for 12 months. At the end of that period, the post-intervention data were collected. No control group was used due to financial constraints. Data prior to the anti-stigma campaign (pre-stigma) were collected in March 2015 (Visit 1). The anti-stigma campaign was delivered from the middle of March until the end of June 2015. Visit-2 data (post-stigma) were collected immediately after the stigma campaign ended in June–July 2015. Next, the interventions using mobile technology-enabled mental health services were delivered by primary healthcare workers and doctors over 12 months. Post-intervention data, including only those participants with an identifiable CMD, were then collected from all of the participating villages. In addition, we collected stigma-related information primarily between March and April 2017 from all adults in the two villages in which the pre-stigma data collection had been done, and this constituted the Visit-3 data (post-intervention). Thus, the time between Visit 1 and Visit 3 was about 24 months. Between Visit 2 and Visit 3 the anti-stigma campaign was not implemented as such, but mental health services were delivered by primary healthcare workers and doctors using mobile technology and this provided exposure to mental health in the community in the form of screening, diagnosis and follow-up.

Ethical approval for the study was obtained from the Independent Ethics Committee of the Centre for Chronic Disease Control, New Delhi (CCDC_IEC_03_2014). Written informed consent was obtained from all participants. The study was conducted as per the Declaration of Helsinki of 1975, as revised in 2008. Data were reported as per the Strengthening the Reporting of Observational Studies in Epidemiology (STROBE) guidelines for reporting observational studies.^9^

### Components of the anti-stigma campaign

An initial literature search was conducted to identify high quality anti-stigma interventions related to mental health and HIV which had been conducted in India or other LMICs. An initial set of strategies was conceived which primarily included printed materials and brochures. These were tested through formative research and additional methods of delivering the campaign were identified, such as using drama and tailoring terms to the locally understandable language (Telugu) to describe stress and depression.^6^

The final strategies implemented in Telugu were:

(a) printed information, education and communication materials. This involved developing brochures, pamphlets and posters on signs and symptoms of CMD such as depression, suicidal risk, stress and how they differed from severe mental disorders; the need for seeking treatment and how it could affect health; and issues of stigma related to mental health which are prevalent in the community. Vignettes on CMD were included in the brochures as examples and discussed. These were shown and discussed via door-to-door campaigns three to four times during the 3-month period. Posters on CMDs, treatment and general awareness about mental disorders were also made available at public places such as schools, administrative buildings and primary health centres.
(b) a video of a person with CMD talking about their experience. An individual and his caretaker were filmed talking about their experience with CMD. This video was shown to everyone during the door-to-door campaign as an example of social contact.
(c) a promotional video on mental health, stigma and the SMART Mental Health project. A local film actor helped promote the SMART Mental Health project and the need to get treated for CMDs in a short video.
(d) drama by a local theatre group on CMD and help seeking. A local theatre group staged a drama on domestic violence, its impact on mental health and the need to seek treatment. This was staged live or shown as a video recording across all villages.

### Instruments used for the quantitative evaluation

Quantitative data were collected at all three time points by trained interviewers using 7-inch Android tablets. At the outset, the interviewers clarified the context and stressed that the anti-stigma campaign was specifically related to CMD and so was the assessment. The key instruments used for measuring stigma and mental health awareness were:

(a) Barriers to Access to Care Evaluation: Treatment Stigma subscale (BACE-TS version 3).^10^ This is a 12-item questionnaire with a four-point Likert scale (‘not at all’, ‘a little’, ‘quite a lot’ or ‘a lot’ which were scored 0, 1, 2 or 3, respectively) which asks questions on the stigma associated with seeking help for mental illnesses. Higher scores suggest higher stigma. BACE has moderate to good reliability and good construct validity.^11^ The questionnaire was translated to Telugu and back translated by independent experts, and no differences were identified. The test-retest reliability using a standardised Cronbach's *α*-test was 0.85 (*N* = 1348), indicating good internal consistency.^8^
(b) Mental Health Knowledge, Attitude and Behaviour (KAB).^12^ This is a 16-item questionnaire^12^ based on other tools, and 12 of those items ascertain mental health knowledge, attitude and behaviours as per the framework for understanding stigma.^1^ The behaviour subgroup was based on the Reported and Intended Behaviour Scale^13^ and summary scores for that subgroup could be generated. This instrument uses a five-point Likert scale (ranging from ‘agree strongly’ which scored 1, to ‘disagree strongly’ which scored 5). Higher scores suggest increased stigma, except for those questions which had a negative connotation (‘mentally ill people tend to be violent’, ‘people with mental illness cannot live a good, rewarding life’, ‘mentally ill people shouldn't get married’ and ‘people with mental health problems should not be given any responsibility’). The instrument was translated to Telugu and back translated into English, by independent experts. The knowledge and attitude subgroups were identified based on discussion with experts (G.T. and M.K.) rather than by any psychometric analyses and hence did not have the properties of a scale.

### Data management and analysis

This was an exploratory pilot study to establish appropriate sample sizes for future studies, so no *a priori* sample-size estimates were computed for these outcomes. Every available consenting adult in the two villages, at each time point, were interviewed. However, only data pertaining to those who were interviewed at all three time points are included for analyses in this article. The primary objective was to understand how the outcomes varied in the same cohort over time. All data were captured electronically and stored on secure servers based in the George Institute for Global Health, India. A statistical plan was developed prior to analysing the data. The mean scores on each item of KAB and BACE-TS were computed. Paired *t*-tests were used to estimate statistical significance between differences in mean scores between Visit 2 and Visit 1, between Visit 3 and Visit 2 and between Visit 3 and Visit 1. A similar analytical plan was executed for the BACE-TS items. We also conducted *post hoc* analyses to check if there were any overall differences by gender (male/female) and education (educated until primary level/educated above primary level).

## Results

The total number of people who were interviewed at Visits 1, 2 and 3 were 1576, 2100 and 1864, respectively. However, 1417 of these people were interviewed at all three visits, and all subsequent longitudinal analyses has focused on those participants. About 10% of those interviewed at Visit 1 were lost to follow-up at one subsequent visit. The sociodemographic profile of the population in the two villages included in the evaluation was similar to the larger set of 12 villages which were part of the SMART Mental Health project. The sociodemographic characteristics of the two villages at baseline were also similar (Supplementary Table 1, available at https://doi.org/10.1192/bjp.2018.190). The mean age (~40 years), gender distribution (~60% female), education (~30% with no schooling), marital status (~80% married) and occupation (~35% being housewife/retired) were similar (data not shown).^8^ The sociodemographic characteristics of the participants at all three visits were also similar (Table 1), as were the sociodemographic characteristics of those who were interviewed at all three visits and those lost to follow-up (Supplementary Table 2).

**Table 1:** Sociodemographic characteristics of the study participants

Characteristics	Visit 1 (pre-stigma; *N* = 1576)	Visit 2 (post-stigma; *N* = 2100)	Visit 3 (post-intervention; *N* = 1864)
	*n* (%)		
Gender			
Female	929 (58.9%)	1150 (54.8%)	1040 (55.8%)
Male	647 (41.1%)	950 (45.2%)	824 (44.2%)
Occupation			
House wife/retired	612 (38.8%)	559 (26.6%)	607 (35.7%)
Unorganised sector	785 (49.8%)	1133 (54.0%)	955 (56.2%)
Organised sector	40 (2.5%)	44 (2.1%)	58 (3.4%)
Other	139 (8.8%)	364 (17.3%)	80 (4.7%)
Education			
No school	507 (32.2%)	640 (30.5%)	456 (26.8%)
Primary school	746 (47.3%)	934 (44.5%)	795 (46.8%)
High school	267 (16.9%)	422 (20.1%)	366 (21.5%)
Graduate/postgraduate	49 (3.1%)	84 (4.0%)	60 (3.5%)
Other	7 (0.4%)	20 (1.0%)	23 (1.4%)
Marital status			
Currently married	1261 (80.0%)	1703 (81.1%)	1499 (80.4%)
Never married	151 (9.6%)	199 (9.5%)	150 (8.0%)
Separated/divorced/ widowed	164 (10.4%)	198 (9.4%)	215 (11.5%)
Age (years)			
18–29	374 (23.7%)	543 (25.9%)	454 (24.4%)
30–49	693 (44.0%)	930 (44.3%)	857 (46.0%)
50–69	405 (25.7%)	499 (23.8%)	450 (24.1%)
≥70	104 (6.6%)	128 (6.1%)	103 (5.5%)
Mean (s.d.)	42.8 (15.79)	41.8 (15.65)	41.9 (15.27)
Range	18; 90	18; 90	18; 90

*N*, total number of participants in each visit; *n*, number of participants with particular characteristic.

### KAB

Most of the items on this questionnaire showed improvement across all visits. Within the knowledge domain, the item related to ‘mentally ill people tend to be violent’ worsened slightly but the change was not statistically significant. Additionally, the item ‘people with mental illness cannot live a good, rewarding life’ was worse at Visit 3 when compared with Visit 1, but had shown significant improvement between Visit 2 and Visit 3 (*P* < 0.001). The attitude-related item ‘people with mental health problems should not be given any responsibility’ worsened at Visit 2 but showed improvement later at Visit 3; although when compared with Visit 1, the scores at Visit 3 were significantly worse (*P* < 0.001) (Table 2). The overall trends for the knowledge, attitude and behaviour components when stratified by gender or education were similar (Supplementary Figures 1–3).

**Table 2:** Changes in mean scores of Knowledge Attitude and Behaviour questions (*N* = 1417)

Category	Question	Visit 1 (pre-stigma)	Visit 2 (post-stigma)	Visit 3 (post-intervention)	Visit 2 − Visit 1	Visit 3 − Visit 2	Visit 3 − Visit 1
		Mean (s.d.)			Difference in mean (s.d), *P*-value		
Knowledge	Mentally ill people tend to be violent	2.3 (1.15)	2.2 (1.23)	2.3 (1.37)	−0.059 (1.675), 0.19	0.033 (1.796), 0.49	−0.025 (1.762), 0.59
	People with mental illness cannot live a good, rewarding life	2.2 (1.03)	1.8 (1.02)	2.1 (1.35)	−0.423 (1.478), <0.001	0.298 (1.695), <0.001	−0.125 (1.681), 0.005
	People with severe mental health problems can fully recover	1.9 (0.94)	1.7 (0.96)	1.6 (0.93)	−0.131 (1.291), <0.001	−0.147 (1.277), <0.001	−0.279 (1.286), <0.001
	Medication can be an effective treatment for people with mental health problems	1.7 (0.93)	1.6 (0.90)	1.3 (0.72)	−0.102 (1.267), 0.002	−0.229 (1.108), <0.001	−0.331 (1.115), <0.001
Attitude	Mentally ill people shouldn't get married	2.4 (1.24)	2.5 (1.40)	2.7 (1.51)	0.091 (1.801), 0.058	0.243 (2.014), <0.001	0.335 (1.941), <0.001
	People with mental health problems are far less of a danger than most people suppose	2.1 (1.00)	1.7 (0.88)	1.6 (1.02)	−0.445 (1.307), <0.001	−0.126 (1.354), <0.001	−0.571 (1.405), <0.001
	We need to adopt a far more tolerant attitude towards people with mental illness in our society	1.6 (0.85)	1.3 (0.62)	1.1 (0.34)	−0.282 (1.047), <0.001	−0.204 (0.701), <0.001	−0.486 (0.89), <0.001
	People with mental health problems should not be given any responsibility	2.3 (1.19)	1.9 (1.18)	2.2 (1.43)	−0.368 (1.637), <0.001	0.258 (1.805), <0.001	−0.11 (1.865), 0.026
Behaviour	I would be willing to live with someone with a mental health problem	2.0 (1.06)	1.7 (1.06)	1.1 (0.61)	−0.337 (1.436), <0.001	−0.516 (1.201), <0.001	−0.853 (1.224), <0.001
	I would be willing to work with someone with a mental health problem	2.0 (1.1)	1.6 (1.03)	1.3 (0.76)	−0.363 (1.397), <0.001	−0.368 (1.225), <0.001	−0.730 (1.304), <0.001
	I would be willing to live nearby someone with a mental health problem	2.0 (1.08)	1.6 (1.00)	1.3 (0.76)	−0.391 (1.405), <0.001	−0.334 (1.189), <0.001	−0.725 (1.297), <0.001
	I would be willing to continue a relationship with a friend who developed a mental health problem	1.9 (1.01)	1.6 (0.93)	1.2 (0.71)	−0.299 (1.317), <0.001	−0.323 (1.141), <0.001	−0.622 (1.212), <0.001
	Overall behaviours score^a^	1.98 (0.94)	1.63 (0.9)	1.23 (0.60)	−0.348 (1.215), <0.001	−0.393 (1.036), <0.001	−0.741 (1.089), <0.001

*P*-values are calculated using paired *t*-tests and will only include participants who had responses for the mentioned questions at both visits.

a This is the average of average individual behaviour scores. The average individual behaviour score was calculated by the sum of all behaviour scores divided by the number of behavioural questions to which that individual responded.

The changes in summary behaviour scores between Visit 3 and Visit 1 were compared by gender and education level. For 569 males and 848 females who were interviewed at both visits, the difference was −0.7 and −0.8, respectively, and both differences were significant (*P* < 0.001). For the 957 individuals with education until primary level and 460 individuals with above primary education level who were interviewed at both visits, the difference was −0.8 and −0.7, respectively, and both differences were significant (*P* < 0.001) (Supplementary Table 3).

### BACE-TS

All items on the BACE-TS showed significant improvement over each time point (Table 3). The overall trend by gender or education level was similar (Supplementary Figure 4).

**Table 3:** Change in mean scores of Barriers to Access to Care Evaluation questions

Items in questionnaire	Visit 1 (pre-stigma)	Visit 2 (post-stigma)	Visit 3 (post- intervention)	Visit 2 − Visit 1	Visit 3 − Visit 2	Visit 3 − Visit 1
	*n*, mean (s.d.)			*n*, difference in mean (s.d.), *P*-value		
Concern that I might be seen as weak for having a mental health problem	1417, 0.38 (0.67)	1223, 0.16 (0.39)	1417, 0.03 (0.18)	1223, −0.229 (0.755), <0.001	1223, −0.128 (0.437), <0.001	1417, −0.354 (0.688), <0.001
Concern that it might harm my chances when applying for jobs	307, 0.65 (0.81)	485, 0.09 (0.3)	1123, 0.03 (0.19)	130, −0.385 (0.811), <0.001	437, −0.064 (0.372), <0.001	246, −0.569 (0.814), <0.001
Concern about what my family might think, say, do or feel	1417, 0.41 (0.66)	1223, 0.18 (0.47)	1417, 0.02 (0.17)	1223, −0.233 (0.801), <0.001	1223, −0.161 (0.498), <0.001	1417, −0.393 (0.673), <0.001
Feeing embarrassed or ashamed	1417, 0.36 (0.64)	1223, 0.19 (0.41)	1417, 0.01 (0.14)	1223, −0.170 (0.744), <0.001	1223, −0.173 (0.431), <0.001	1417, −0.347 (0.658), <0.001
Concern that I might be seen as ‘crazy’	1417, 0.35 (0.68)	1223, 0.13 (0.36)	1417, 0.02 (0.19)	1223, −0.227 (0.756), <0.001	1223, −0.103 (0.412), <0.001	1417, −0.333 (0.694), <0.001
Concern that I might be seen as a bad parent	1417, 0.36 (0.65)	1105, 0.16 (0.38)	1288, 0.01 (0.12)	1105, −0.217 (0.742), <0.001	1073, −0.152 (0.41), <0.001	1288, −0.374 (0.676), <0.001
Concern that people I know might find out	1417, 0.39 (0.65)	1223, 0.13 (0.35)	1417, 0.02 (0.15)	1223, −0.247 (0.687), <0.001	1223, −0.115 (0.385), <0.001	1417, −0.372 (0.659), <0.001
Concern that people might not take me seriously if they found out I was having professional care	1417, 0.41 (0.66)	1223, 0.13 (0.44)	1417, 0.01 (0.14)	1223, −0.284 (0.752), <0.001	1223, −0.114 (0.461), <0.001	1417, −0.394 (0.675), <0.001
Not wanting a mental health problem to be on my medical records	1417, 0.32 (0.75)	1223, 0.04 (0.21)	1417, 0.03 (0.24)	1223, −0.278 (0.782), <0.001	1223, −0.012 (0.325), 0.187	1417, −0.284 (0.787), <0.001
Concern that my children may be taken into care or that I may lose access or custody without my consent	1417, 0.38 (0.72)	1100, 0.19 (0.41)	1288, 0.02 (0.21)	1100, −0.205 (0.828), <0.001	1068, −0.175 (0.469), <0.001	1288, −0.381 (0.767), <0.001
Concern about what my friends might think, say or do	1417, 0.44 (0.69)	1223, 0.14 (0.37)	1417, 0.01 (0.13)	1223, −0.292 (0.773), <0.001	1223, −0.132 (0.394), <0.001	1417, −0.423 (0.697), <0.001
Concern about what people at work might think, say or do	1417, 0.45 (0.71)	1223, 0.22 (0.53)	1417, 0.01 (0.15)	1223, −0.224 (0.873), <0.001	1223, −0.206 (0.557), <0.001	1417, −0.436 (0.726), <0.001
Overall BACE score^a^	1417, 0.39 (0.51)	1223, 0.15 (0.25)	1417, 0.02 (0.13)	1223, −0.242 (0.539), <0.001	1223, −0.133 (0.286), <0.001	1417, −0.375 (0.519), <0.001

*P*-values are calculated using paired *t*-tests and will only include participants who had responses for the mentioned questions at both visits.

a This is the average of average individual BACE scores. The average individual BACE score was calculated by the sum of all scores divided by the number of questions to which that individual responded.

The changes in total BACE scores between Visit 3 and Visit 1 were compared by gender and education level. Overall mean scores on stigma perceptions improved by −0.375 (minimum/maximum of −2.7/2.4, s.d. 0.519, *P* < 0.001). For the 569 males and 848 females who were interviewed at both visits, the difference was −0.3 and −0.4, respectively, and both differences were significant (*P* < 0.001). For the 957 individuals with primary level education and 460 individuals with above primary level education who were interviewed at both visits, the difference was −0.4 and −0.3, respectively, and both differences were significant (*P* < 0.001) (Supplementary Table 4).

## Discussion

This study reports on the longitudinal changes in knowledge, attitude and behaviour related to mental health and perceptions on seeking help for mental illness following a rural community-based study. To the best of our knowledge, this is the first study from an LMIC that reports on the longitudinal impact of an anti-stigma campaign in a large community-based population. Earlier research from India which delivered a mental health awareness intervention for CMDs in the community have only reported on cross-sectional outcomes, and have also used strategies that are different from those we have implemented.^14^^,^^15^ Although SMART Mental Health and both of the earlier two projects^14^^,^^15^ used broadly similar intervention strategies (brochures, home visits, drama and movie), they differed in their implementation. First, our use of a ‘social contact’ (in the form of a video of a person with mental illness narrating their experience) did not seem to have been used explicitly by the other two projects. Social contact is perceived as the most effective intervention for an anti-stigma campaign,^2^ in agreement with our observations.^8^ Second, in this article we are reporting the longitudinal outcomes of the intervention, whereas the other two studies only provide cross-sectional data.

The results suggest that over the 2-year follow-up period the study participants had generally shown sustained improvement in knowledge, attitude and behaviour towards mental health (barring three items), and perceptions of stigma related to seeking help were reduced. This benefit was apparent despite the fact that the intensive anti-stigma campaign was conducted only in the first 3 months, after which the intervention involving the delivery of mental health services was implemented for 12 months. Our previous report, based on data collected soon after the intensive campaign was delivered, showed improvements in attitudes, behaviours and perceptions related to seeking help and we found that these have continued to improve even further, and knowledge about mental health has also improved. This underlines the sustained impact of the intervention over a long period of time.

Availability of data from LMICs is limited and availability of longitudinal data (apart from this study) from LMICs is absent.^16^ Even for the three items that scored worse at follow-up, the trends suggest that the scores were improving after an initial drop immediately following the anti-stigma campaign. It could be that, over time, these negative attitudes reversed due to a better understanding of mental health issues. However, it is not possible to infer if the model used for the delivery of mental health services had an impact on stigma and mental health knowledge, attitude and behaviour *per se*; it is possible that availability of community-based mental health services over the 12-month period may have led to positive changes due to increased awareness in the community. This could explain the almost tenfold drop in stigma perceptions related to service use as against a more modest change in knowledge, attitude and behaviour. As opined earlier,^8^ the slightly negative perception that people with mental illness cannot lead a good, rewarding life could be due to how people interpreted the question in light of overall quality of life rather than a situation where you live a ‘good, rewarding life’ with some residual disability. The negative perception that ‘people with mental health problems should not be given any responsibility’ could be the general belief that people with any illness (including mental illness) need rest and should not be burdened with responsibility. Both of these perceptions need further clarification in future research.

When the differences in total behaviour score and total BACE scores were stratified by gender and education level, significant differences towards improved scores were observed at 2 years, irrespective of gender or education level. When total behaviour scores were compared by gender at Visit 1 and Visit 3, it was seen that women had a slightly higher score at both time points (poorer behaviour scores) compared with men, and the difference was significant at Visit 1 but not at Visit 3. However, the perceptions to seeking help (total BACE scores) were not significantly different across genders at either time points (Supplementary Tables 3 and 4).

Those with education up to primary level had worse scores on both the behaviour scale and help-seeking (BACE) scale at both time points compared with those with higher level education, and the difference was significant (*P* < 0.001) at Visit 1 but not at Visit 3. Corrigan and Watson^17^ had reported differences in stigma perception based on gender and education (with less perception of stigma among women and those with increased education); our study suggested the opposite results for gender, but similar results for education level. However, at follow-up at Visit 3, the differences were non-significant for both gender and education, making it difficult to draw any firm conclusion. More research is needed on populations from LMICs to understand the effect of gender and education on perceptions of stigma related to seeking help and behaviour related to mental health.

The study design is limited by not having a control group, hence the results need to be interpreted with caution. We are thus unable to comment on effectiveness of the intervention or its definitive impact over the 2-year period. However, the results in this article are derived from the repeated-measures design, where the participants were followed for a 2-year period and outcome was assessed at the end of it. This provides an understanding of the long-term impact of the campaign and provides data showing beneficial sustained effect, which should be replicated in future using controlled studies. Although the KAB and BACE had not undergone any psychometric testing for this population, both tools had undergone translation and back translations and the test-retest reliability of the BACE was good. Because of this, and as this was an exploratory pilot study, we have reported only unadjusted changes in scores. There was no *a priori* hypothesis to adjust for confounders or conduct any sophisticated analyses. Finally, although the interviewers always asked respondents to refer to CMD while answering the questions, both KAB and BACE were not specific to severe mental disorders or CMDs and it may thus be possible that some participants still responded in terms of severe mental disorders, leading to information bias.

The clinical and public health implications of these results are not based on the absolute changes in score as they are minimal. However, the importance of this study lies in the sustained improvement in the scores, even after a long period has elapsed following the anti-stigma campaign, and results in some public health implications for policy makers: First, a mental health awareness campaign needs to be integrated into the delivery of routine mental health services. Second, an intensive campaign may be needed for shorter periods of time followed by subsequent booster sessions, and this could also be integrated within the delivery of existing services. Third, a service delivery model that follows a mental health awareness campaign may help to reduce stigma. Some of these implications need to be replicated in other studies as well as tested separately using factorial study designs.

The treatment gap in providing care for people with CMDs in LMICs and the role stigma plays in accessing care are well documented.^18^^–^^20^ Thus, identifying culturally relevant methods to deliver effective anti-stigma campaigns is particularly relevant in the context of countries like India, where stigma is high. The long-term outcome evident from this project suggests that the method used to deliver the anti-stigma campaign was apparently effective and sustainable. Some implications of the campaign were evident in the increased uptake of mental health services and reduction in depression and anxiety scores in 30 of the 42 villages involved in the anti-stigma campaign,^21^ and are also apparent in preliminary results from the remaining set of 12 villages which includes the two villages involved in this study (details available from the author P.K.M. on request). The process evaluation from the set of 30 villages indicates that the intervention is acceptable, feasible and can be scaled up. It has identified a set of barriers and facilitators which need to be addressed.^22^ Future randomised controlled studies can address these issues, add to the evidence and provide data on cost effectiveness so that this methodology could then be applied across similar settings after suitable adaptations.

## Supplementary material

For supplementary material accompanying this paper visit https://doi.org/10.1192/bjp.2018.190. click here to view supplementary material
