# Supplementary material for: Longitudinal assessment of an anti-stigma campaign related to common mental disorders in rural India
Source: Br J Psychiatry. 2019 Feb;214(2):90–5. doi: 10.1192/bjp.2018.190 (PMC6420138; doi:10.1192/bjp.2018.190)
Supplement: Supplementary file 1 [file S0007125018001903sup001.docx]

**Supplementary Table 1: Sociodemographic characteristics of the two villages at pre-stigma**

|  | **Visit 1 (Pre-Stigma) (N=1576)** | | | |
| --- | --- | --- | --- | --- |
|  | | **Comparison between two village** | | |
|  | **Total (N=1576)** | **Ravulaparru (N=731)** | **Vallurupalli (N=845)** |  |
| **Characteristic** | **n (%)** | **n (%)** | **n (%)** |  |
| Gender |  |  |  |  |
| Female | 929 (58.9%) | 424 (58.0%) | 505 (59.8%) |  |
| Male | 647 (41.1%) | 307 (42.0%) | 340 (40.2%) |  |
|  |  |  |  |  |
| Occupation |  |  |  |  |
| House wife/Retired | 612 (38.8%) | 202 (27.6%) | 410 (48.5%) |  |
| Organized Sector | 40 (2.5%) | 16 (2.2%) | 24 (2.8%) |  |
| Other | 139 (8.8%) | 68 (9.3%) | 71 (8.4%) |  |
| Unorganized Sector | 785 (49.8%) | 445 (60.9%) | 340 (40.2%) |  |
|  |  |  |  |  |
| Education |  |  |  |  |
| Graduate/Post Graduate | 49 (3.1%) | 18 (2.5%) | 31 (3.7%) |  |
| High School | 267 (16.9%) | 94 (12.9%) | 173 (20.5%) |  |
| No School | 507 (32.2%) | 293 (40.1%) | 214 (25.3%) |  |
| Other | 7 (0.4%) | 2 (0.3%) | 5 (0.6%) |  |
| Primary School | 746 (47.3%) | 324 (44.3%) | 422 (49.9%) |  |
|  |  |  |  |  |
| Marital Status |  |  |  |  |
| Currently Married | 1261 (80.0%) | 592 (81.0%) | 669 (79.2%) |  |
| Never Married | 151 (9.6%) | 59 (8.1%) | 92 (10.9%) |  |
| Separated/Divorced/Widowed | 164 (10.4%) | 80 (10.9%) | 84 (9.9%) |  |
|  |  |  |  |  |
| Age(Years) |  |  |  |  |
| Mean (SD) | 42.8 (15.79) | 43.9 (16.21) | 41.8 (15.36) |  |
| Range | 18; 90 | 18; 86 | 18; 90 |  |

*N=Total number of patients in that visit. n=Number of patients with particular characteristic.*

**Supplementary Table 2: Differences in sociodemographic characteristics amongst those interviewed at pre-stigma and those followed up for subsequent visits**

|  | **Visit 1 (Pre-stigma) (N=1576)** | | |
| --- | --- | --- | --- |
|  | **Comparison between common subject between all 3 visits and those not followed up** | | |
|  | **Common subjects for all 3 visits (N=1417)** | **Remaining subjects (N=159)** |  |
| **Characteristic** | **n (%)** | **n (%)** | **P-value** |
| Gender |  |  | 0.2861 |
| Female | 829 (58.5%) | 100 (62.9%) |  |
| Male | 588 (41.5%) | 59 (37.1%) |  |
|  |  |  |  |
| Occupation |  |  | 0.8727 |
| House wife/Retired | 553 (39.0%) | 59 (37.1%) |  |
| Organized Sector | 35 (2.5%) | 5 (3.1%) |  |
| Other | 123 (8.7%) | 16 (10.1%) |  |
| Unorganized Sector | 706 (49.8%) | 79 (49.7%) |  |
|  |  |  |  |
| Education |  |  | 0.0025 |
| Graduate/Post Graduate | 39 (2.8%) | 10 (6.3%) |  |
| High School | 228 (16.1%) | 39 (24.5%) |  |
| No School | 456 (32.2%) | 51 (32.1%) |  |
| Other | 7 (0.5%) | 0 (0.0%) |  |
| Primary School | 687 (48.5%) | 59 (37.1%) |  |
|  |  |  |  |
| Marital Status |  |  | 0.3091 |
| Currently Married | 1141 (80.5%) | 120 (75.5%) |  |
| Never Married | 133 (9.4%) | 18 (11.3%) |  |
| Separated/Divorced/Widowed | 143 (10.1%) | 21 (13.2%) |  |
|  |  |  |  |
| Age(Years) |  |  |  |
| Mean (SD) | 43.1 (15.80) | 40.2 (15.48) | 0.0321 |
| Range | 18; 90 | 18; 80 |  |

*N=Total number of patients in that visit. n=Number of patients with particular characteristic.*

**Supplementary Table 3: Change in overall mean Behaviour scores of Knowledge Attitude and Behaviour**

| **Order** | **Overall Behaviour SCORE** | **Group 1** | **Group 2** | **Mean difference (CI)** | **P-Value** |
| --- | --- | --- | --- | --- | --- |
| 1 | **Comparison between Pre-Stigma and Post Intervention** |  |  |  |  |
|  | N | 1417 | 1417 |  |  |
|  | Mean (SD) | 1.98 (0.939) | 1.23 (0.603) | -0.7 (-0.8;-0.7) | <.001 |
| 2 | **For Pre-Stigma only: comparison between Male and Female** |  |  |  |  |
|  | N | 569 | 848 |  |  |
|  | Mean (SD) | 1.91 (0.896) | 2.02 (0.965) | 0.1 (0.0;0.2) | 0.0320 |
| 3 | **For Pre-Stigma only: comparison between Up to primary and above primary** |  |  |  |  |
|  | N | 957 | 460 |  |  |
|  | Mean (SD) | 2.02 (0.939) | 1.89 (0.934) | -0.1 (-0.2;-0.0) | 0.0133 |
| 4 | **For male only: comparison between Pre-Stigma and Post Intervention** |  |  |  |  |
|  | N | 569 | 569 |  |  |
|  | Mean (SD) | 1.91 (0.896) | 1.20 (0.597) | -0.7 (-0.8;-0.6) | <.001 |
| 5 | **For Female only: comparison between Pre-Stigma and Post Intervention** |  |  |  |  |
|  | N | 848 | 848 |  |  |
|  | Mean (SD) | 2.02 (0.965) | 1.26 (0.607) | -0.8 (-0.8;-0.7) | <.001 |
| 6 | **For Up to primary only: comparison between Pre-Stigma and Post Intervention** |  |  |  |  |
|  | N | 957 | 957 |  |  |
|  | Mean (SD) | 2.02 (0.939) | 1.25 (0.610) | -0.8 (-0.8;-0.7) | <.001 |
| 7 | **For above primary only: comparison between Pre-Stigma and Post Intervention** |  |  |  |  |
|  | N | 460 | 460 |  |  |
|  | Mean (SD) | 1.89 (0.934) | 1.20 (0.589) | -0.7 (-0.8;-0.6) | <.001 |
| 8 | **For Post Intervention only: comparison between Male and Female** |  |  |  |  |
|  | N | 569 | 848 |  |  |
|  | Mean (SD) | 1.20 (0.597) | 1.26 (0.607) | 0.1 (-0.0;0.1) | 0.0917 |
| 9 | **For Post Intervention only: comparison between Up to primary and above primary** |  |  |  |  |
|  | N | 957 | 460 |  |  |
|  | Mean (SD) | 1.25 (0.610) | 1.20 (0.589) | -0.1 (-0.1;0.0) | 0.1347 |

*For comparison between Visit 1(Pre-Stigma) and Visit 3(Post Intervention) p-value was computed by paired t-test. For comparison between the category for a specific visit p-value was calculated by independent 2 sample t-test.*

*Analysis was performed on total Behaviour score. This was calculated by summing up all Behaviour scores divided by number of Behavioural question responded by that individual.*

*For order (1,4,5,6,7) : Group 1 = Visit 1 (Pre-Stigma) and Group 2= Visit 3 (Post Intervention).*

*For order (2 and 8) : Group 1 = Male and Group 2= Female.*

*For order (3 and 9) : Group 1 = Up to primary and Group 2= Above primary.*

**Supplementary Table 4: Change in overall total mean scores of BACE**

| **Order** | **Overall Behaviours SCORE** | **Group 1** | **Group 2** | **Mean difference (CI)** | **P-Value** |
| --- | --- | --- | --- | --- | --- |
| 1 | **Comparison between Pre-Stigma and Post Intervention** |  |  |  |  |
|  | N | 1417 | 1417 |  |  |
|  | Mean (SD) | 0.39 (0.506) | 0.02 (0.125) | -0.4 (-0.4;-0.3) | <.001 |
| 2 | **For Pre-Stigma only: comparison between Male and Female** |  |  |  |  |
|  | N | 569 | 848 |  |  |
|  | Mean (SD) | 0.37 (0.481) | 0.41 (0.521) | 0.0 (-0.0;0.1) | 0.1030 |
| 3 | **For Pre-Stigma only: comparison between Up to primary and above primary** |  |  |  |  |
|  | N | 957 | 460 |  |  |
|  | Mean (SD) | 0.42 (0.523) | 0.34 (0.463) | -0.1 (-0.1;-0.0) | 0.0042 |
| 4 | **For male only: comparison between Pre-Stigma and Post Intervention** |  |  |  |  |
|  | N | 569 | 569 |  |  |
|  | Mean (SD) | 0.37 (0.481) | 0.02 (0.113) | -0.3 (-0.4;-0.3) | <.001 |
| 5 | **For Female only: comparison between Pre-Stigma and Post Intervention** |  |  |  |  |
|  | N | 848 | 848 |  |  |
|  | Mean (SD) | 0.41 (0.521) | 0.02 (0.133) | -0.4 (-0.4;-0.4) | <.001 |
| 6 | **For Up to primary only: comparison between Pre-Stigma and Post Intervention** |  |  |  |  |
|  | N | 957 | 957 |  |  |
|  | Mean (SD) | 0.42 (0.523) | 0.02 (0.132) | -0.4 (-0.4;-0.4) | <.001 |
| 7 | **For above primary only: comparison between Pre-Stigma and Post Intervention** |  |  |  |  |
|  | N | 460 | 460 |  |  |
|  | Mean (SD) | 0.34 (0.463) | 0.02 (0.110) | -0.3 (-0.4;-0.3) | <.001 |
| 8 | **For Post Intervention only: comparison between Male and Female** |  |  |  |  |
|  | N | 569 | 848 |  |  |
|  | Mean (SD) | 0.02 (0.113) | 0.02 (0.133) | 0.0 (-0.0;0.0) | 0.9514 |
| 9 | **For Post Intervention only: comparison between Up to primary and above primary** |  |  |  |  |
|  | N | 957 | 460 |  |  |
|  | Mean (SD) | 0.02 (0.132) | 0.02 (0.110) | 0.0 (-0.0;0.0) | 0.3425 |

*For comparison between Visit 1(Pre-Stigma) and Visit 3(Post Intervention) p-value was computed by paired t-test. For comparison between the category for a specific visit P-value was calculated by independent 2 sample t-test.*

*For order (1,4,5,6,7): Group 1 = Visit 1 (Pre-Stigma) and Group 2= Visit 3 (Post Intervention).*

*For order (2 and 8): Group 1 = Male and Group 2= Female.*

*For order (3 and 9): Group 1 = Up to primary and Group 2= Above primary*

**Supplementary Figure 1: Changes in knowledge across all visits stratified by gender and education**

| **All Subjects** | **Gender = Male** | **Gender = Female** |
| --- | --- | --- |
| **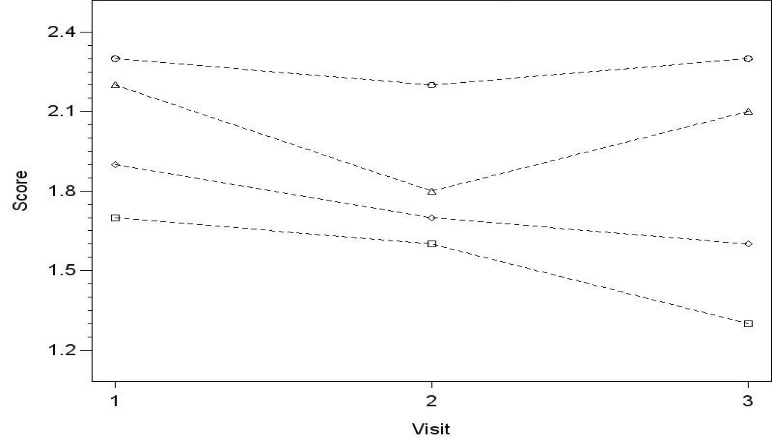** | **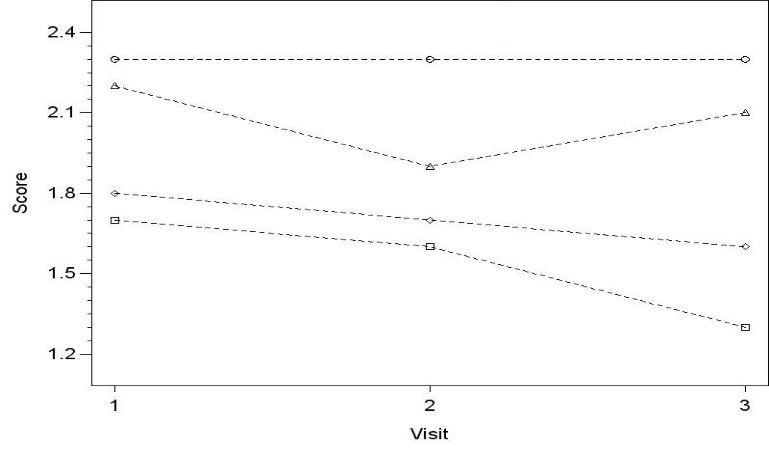** | **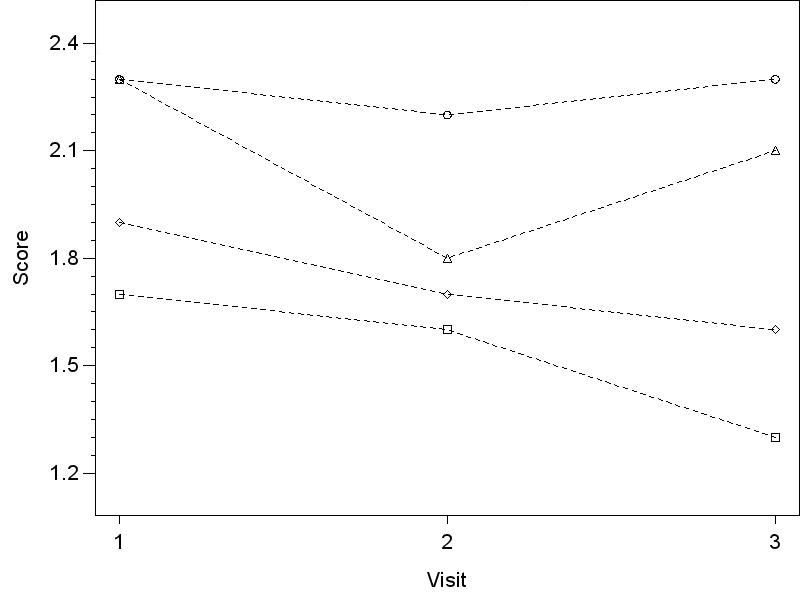** |
| **Symbol definition** | **Education = Up to primary** | **Education = Above primary** |
| **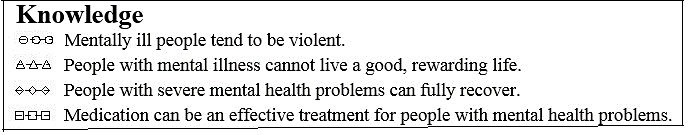** | **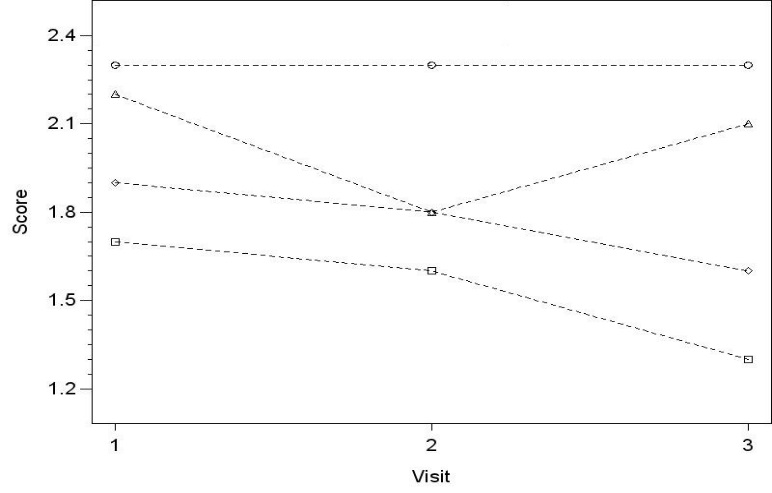** | **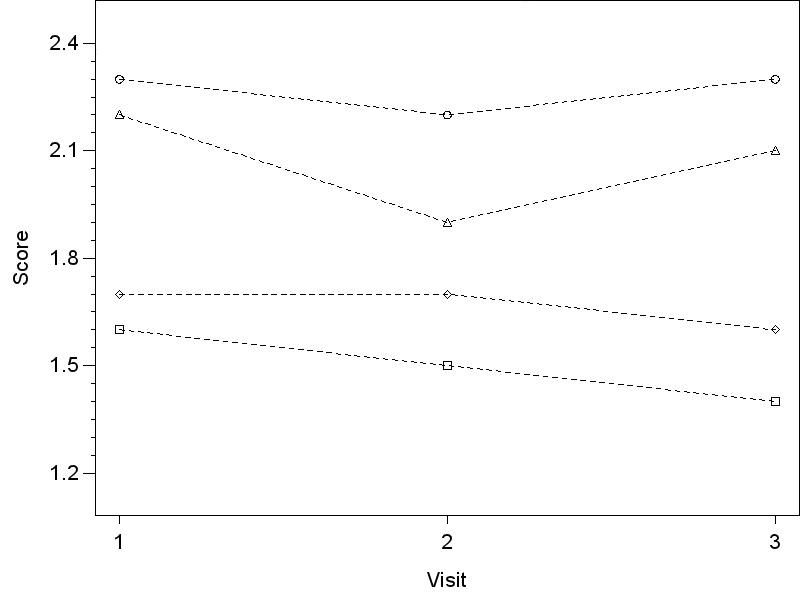** |

**Supplementary Figure 2: Changes in attitude across visits stratified by gender and education**

| **All Subjects** | **Gender = Male** | **Gender = Female** |
| --- | --- | --- |
| **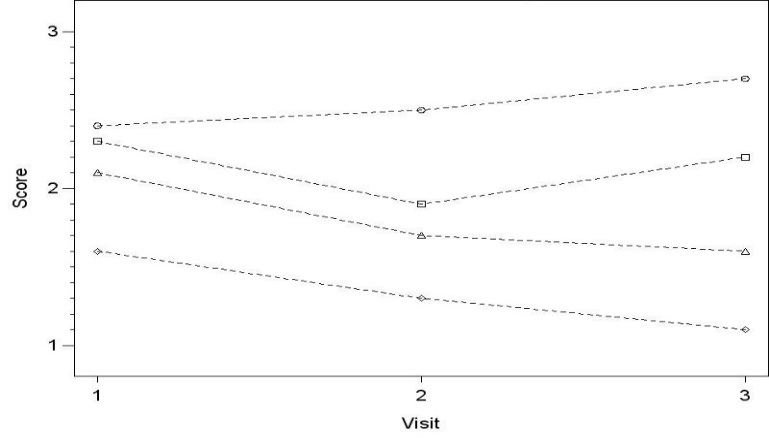** | **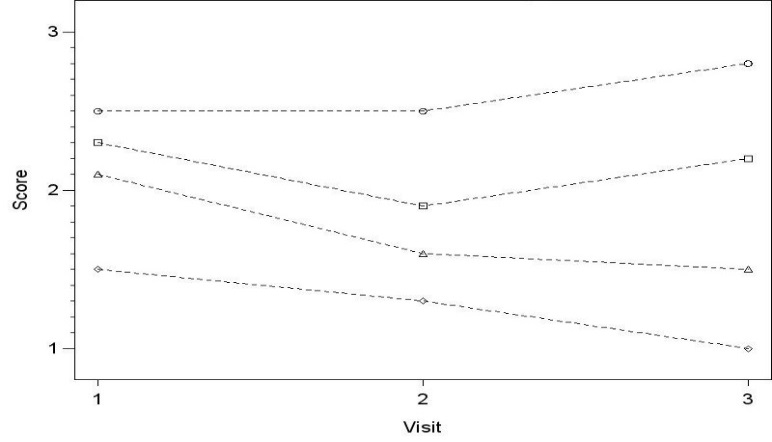** | **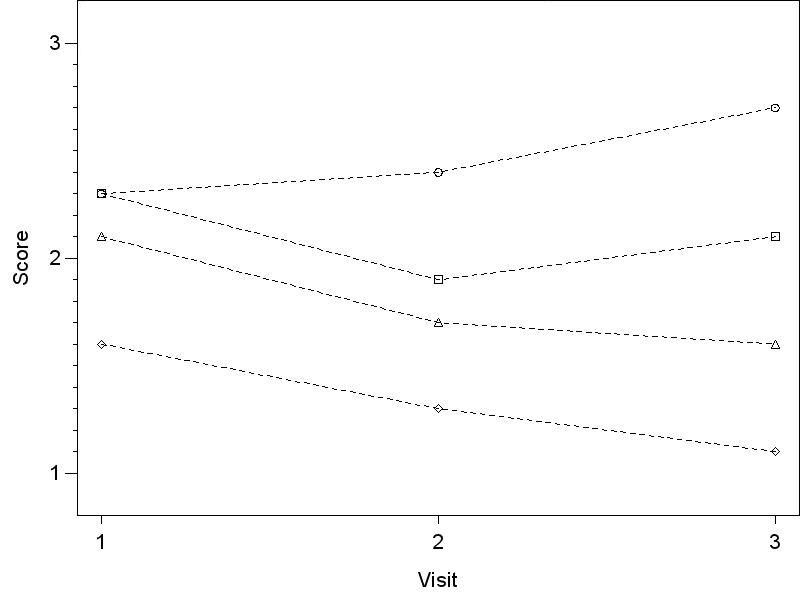** |
| **Symbol definition** | **Education = Up to primary** | **Education = Above primary** |
| 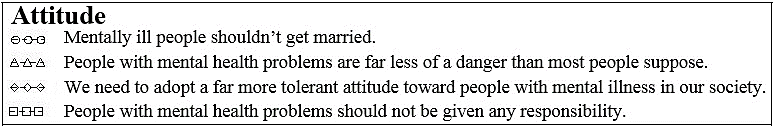 | **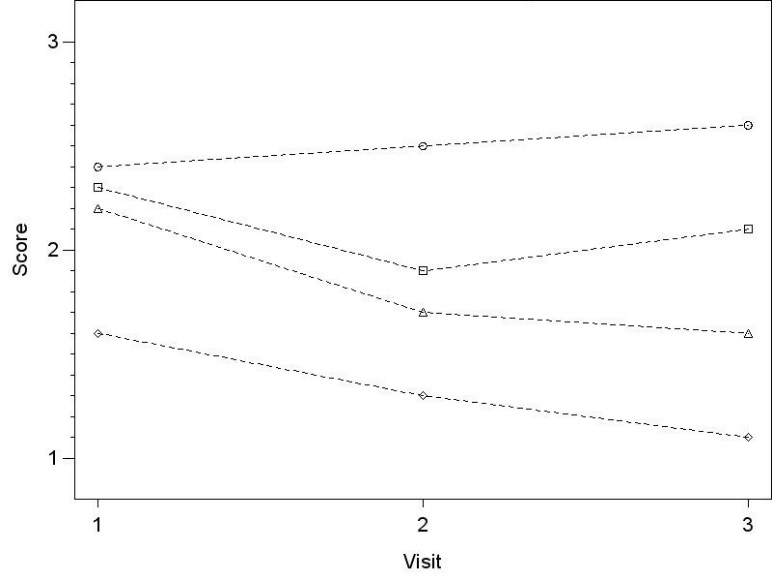** | **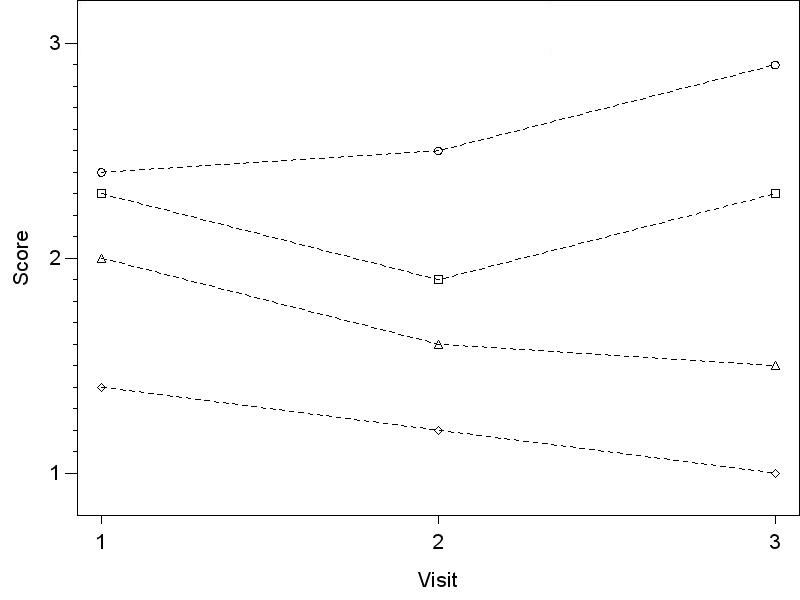** |

**Supplementary Figure 3: Changes in behaviour across visits stratified by gender and education**

| **All Subjects** | **Gender = Male** | **Gender = Female** |
| --- | --- | --- |
| **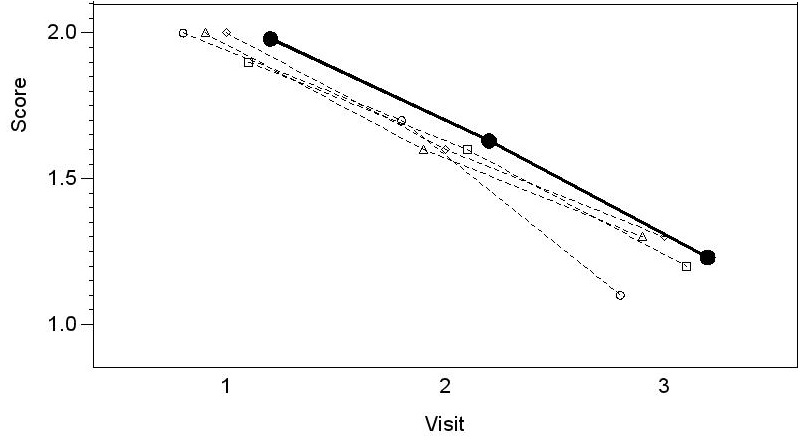** | **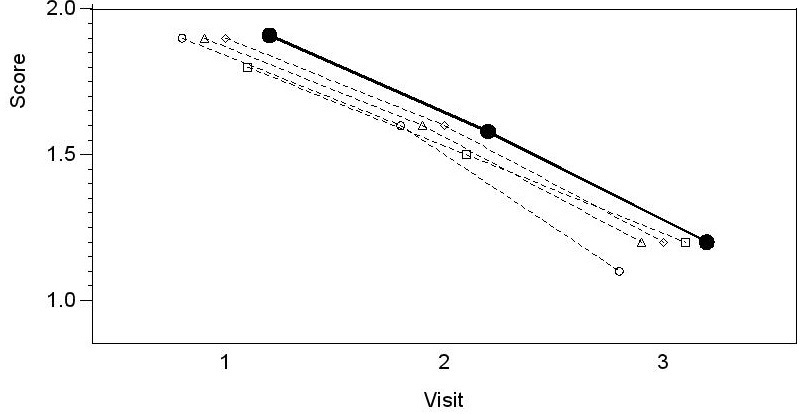** | **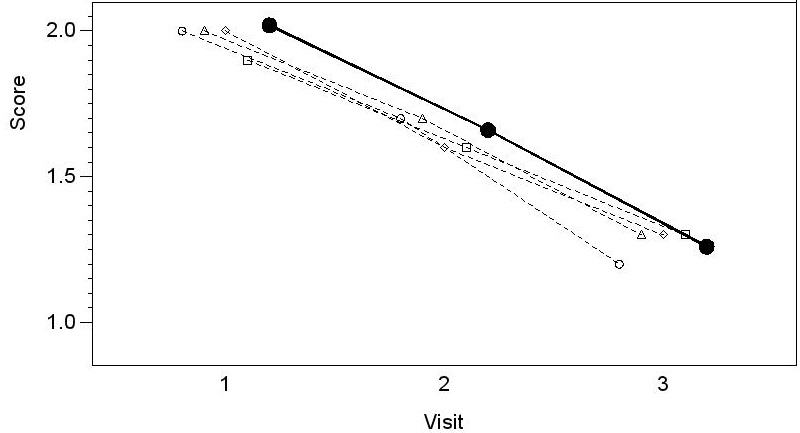** |
| **Symbol definition** | **Education = Up to primary** | **Education = Above primary** |
| **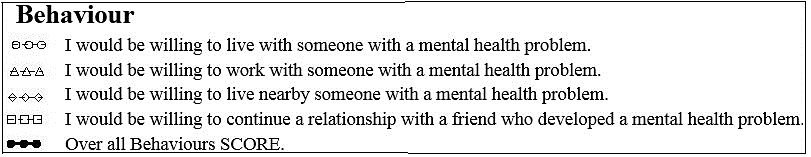** | **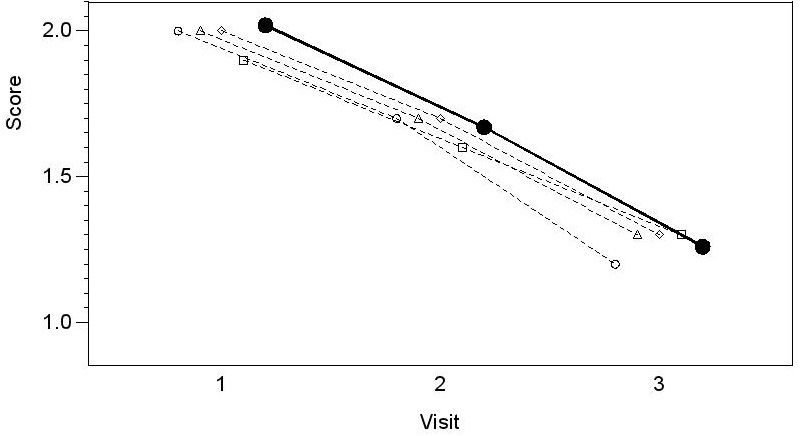** | **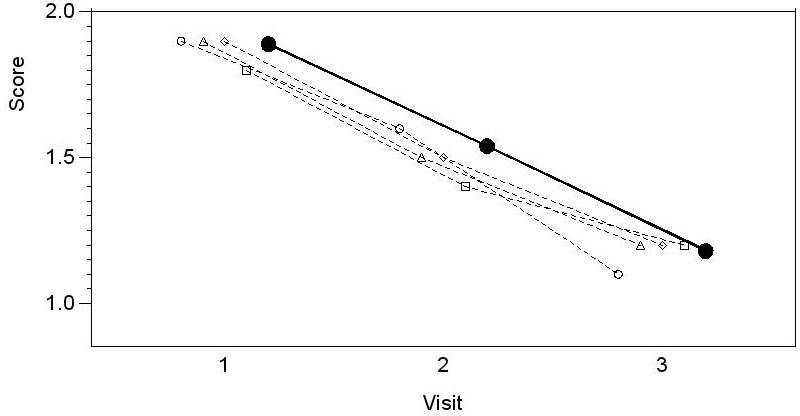** |

**Supplementary Figure 4: Changes in BACE scores across visits stratified by gender and education**

| **All Subjects** | **Gender = Male** | **Gender = Female** |
| --- | --- | --- |
| **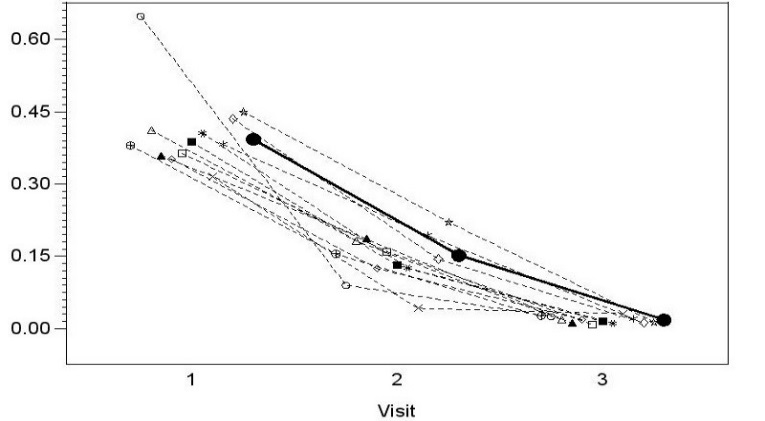** | **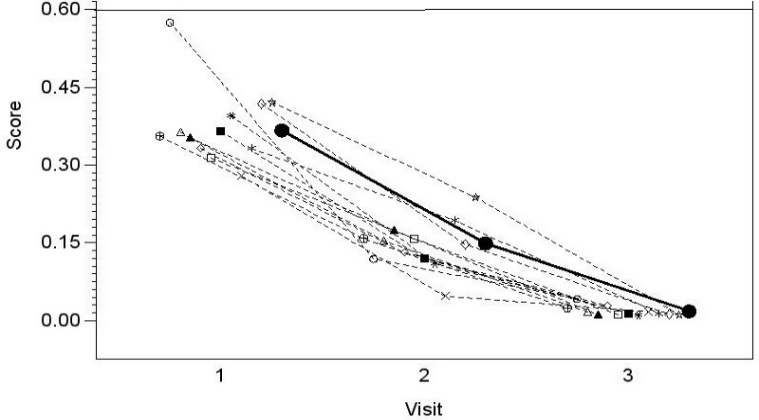** | **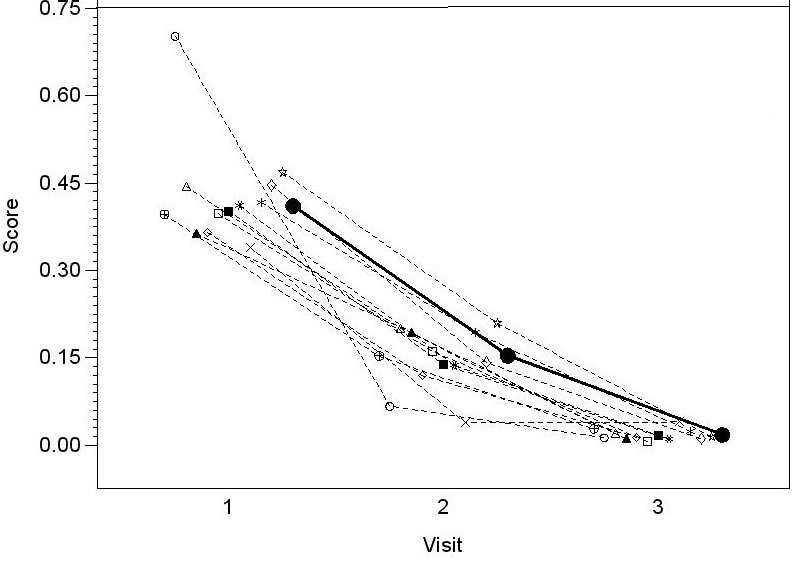** |
|  | **Education = Up to primary** | **Education = Above primary** |
|  | **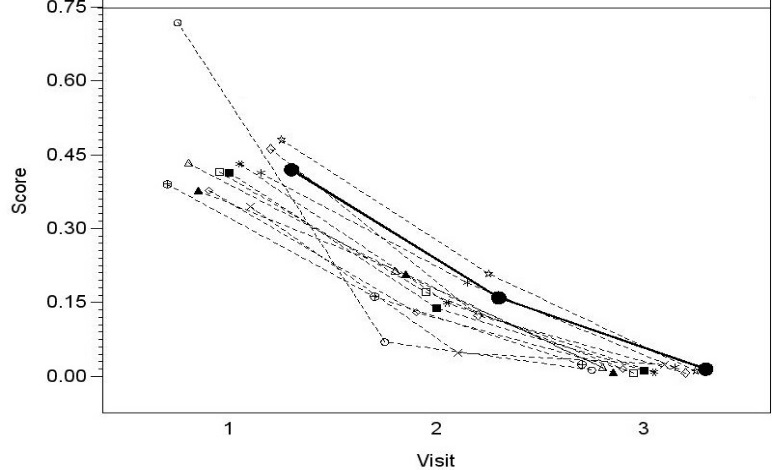** | **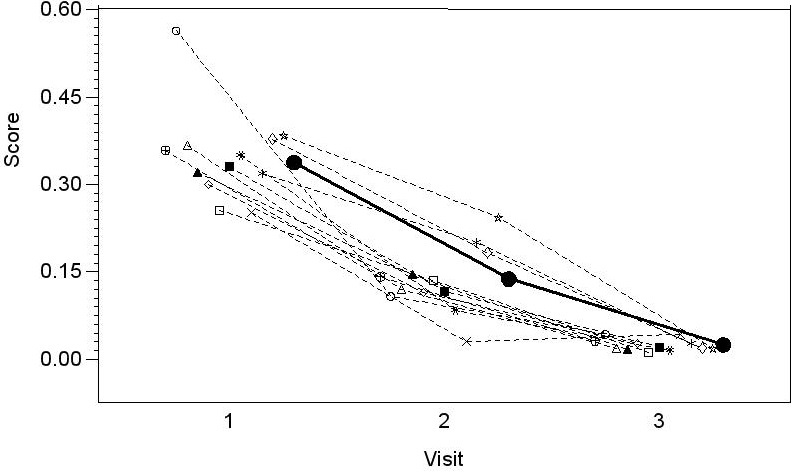** |
| 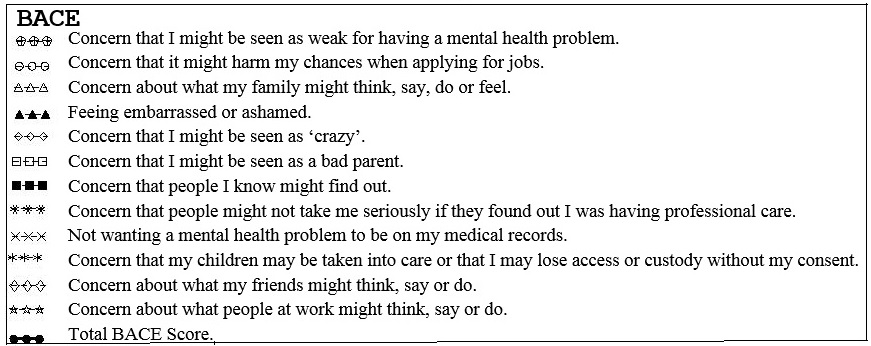 | | |
